# In Vitro Cytotoxicity Assessment of Leaves of *Tieghemella heckelii* on Breast (MDA‐MB‐468), Liver (HepG2), and Prostate (PC3) Cancer Cell Lines

**DOI:** 10.1155/bmri/2002140

**Published:** 2026-03-12

**Authors:** Justice Kumi, Abigail Aning, Janet Ampofo, Sherif Hamidu, Eric N. Y. Nyarko

**Affiliations:** ^1^ Department of Clinical Pathology, Noguchi Memorial Institute for Medical Research, College of Health Sciences, University of Ghana, Accra, Ghana, ug.edu.gh; ^2^ Department of Chemical Pathology, University of Ghana Medical School, College of Health Sciences, University of Ghana, Accra, Ghana, ug.edu.gh

## Abstract

**Introduction:**

Cancer remains the leading cause of death worldwide, with breast cancer being the most prevalent disease diagnosed globally. Liver cancer is the fourth most common cause of death globally, while prostate cancer accounts for the second most frequent malignancy among males worldwide. The main treatment options for cancer include surgery, radiation therapy, and chemotherapy. The stem bark of *Tieghemella heckelii* has been exploited for its medicinal properties. On a broader scale, research on the anticancer properties of *T. heckelii* has not been explored. It is therefore important to investigate the anticancer potential of the leaves of *T. heckelii*. The goal of the study was to evaluate the in vitro anticancer activities of the leaves of *T. heckelii* on breast, prostate, and liver cancer cell lines.

**Methods:**

The leaves of *T. heckelii* were collected from Asantemanso, Akim Oda, in the eastern region of Ghana. Authenticity of the leaves was performed at the Department of Plant and Environmental Biology, University of Ghana. Aqueous and ethanol extraction were performed on the leaves of *T. heckelii* after grinding into fine particles, followed by low‐temperature drying. Breast cancer (MDA‐MB‐468), liver cancer (HepG2), and normal kidney (Vero E6) cell lines were cultured in DMEM media, while prostate cancer (PC3) cell lines were cultured in RPMI medium. Anticancer activity of the leaves of *T. heckelii* was conducted on breast (MDA‐MB‐468), liver (HepG2), and prostate (PC3) cancer cell lines. Cell viability was determined by the 3‐(4,5‐dimethylthiazol‐2‐yl)‐2,5‐diphenyltetrazolium bromide (MTT) method.

**Results:**

Both ethanolic and aqueous extracts demonstrated similar cytotoxicity for the HepG2 cell line, with IC_50_ values of about 200 *μ*g/mL. selectivity index of > 2 was recorded by all cell lines. When compared to other cell lines, the aqueous extract for prostate cancer showed the lowest IC_50_ value with a selectivity of 8. In addition, the extracts showed less cytotoxic activity against the normal (Vero) cell line.

**Conclusion:**

Leaves of *T. heckelii* possess cytotoxic properties with notable selectivity against prostate (PC3) and liver (HepG2) cancer cell lines. However, findings should be evaluated in in vivo studies because biological processes can be more complex in living organisms. Further investigation should be conducted to ascertain the bioactive compounds responsible for the anticancer activity.

## 1. Introduction

Cancer is a diverse group of serious metabolic illnesses characterized by uncontrolled and abnormal cell proliferation that can invade and spread to other parts of the body. Despite improvements in diagnosis, treatment, and prevention, it remains the leading cause of mortality worldwide, with the number of cases steadily increasing and expected to reach 21 million by 2030 [1, 2].

It is estimated that developing countries account for more than half of new cancer cases and two‐thirds of cancer deaths [3]. The cancer epidemic in sub‐Saharan Africa is substantial and anticipated to increase if appropriate measures are not implemented [4]. In Ghana, cancer mortality and incidence rates are on the rise. In 2019, Ghana reported over 95,000 cases of cancer, with estimated records of more than 24,000 new cases annually, with over 15,802 mortalities in 2020 [5].

Breast cancer remains the most prevalent cancer globally [6] and the most frequent cancer in women in Ghana, with a 20.4% incidence and a relatively high mortality rate, according to the World Health Organization (WHO)′s 2020 Cancer Country Profile of Ghana [7].

In 2020, liver cancer was the third leading cause of cancer‐related deaths worldwide [6], fourth in female cancer deaths, and second in male cancer deaths in sub‐Saharan Africa [8]A projected number of 3,731 individuals were diagnosed with new liver cancer cases in 2022, with a mortality rate of 3,362 people in Ghana [9].

Prostate cancer is a major global public health concern and the second most diagnosed cancer in men from sub‐Saharan Africa [6] and the most prevalent cancer diagnosis among men in Ghana, with an annual incidence of 2129 cases and a mortality rate of more than 50% [10].

Treatment options for cancer are normally surgery, radiation therapy, chemotherapy, immunotherapy, hormone therapy, and targeted therapy. However, there are several adverse effects associated with these treatment alternatives, with accessibility and affordability being major issues. Herbal medicine has gained significance in the health industry, mostly due to its perceived natural safety, affordability, cultural alignment, ease of availability, and relatively inexpensive cost [11]. According to the WHO, traditional medicines account for approximately 80% of primary healthcare requirements in developing nations [12]. In Ghana, herbal medicine is an essential component of a healthcare system that is widely available, particularly in rural regions. The government formally recognizes the introduction of herbal medicine therapy in the healthcare system as an integral aspect of Ghana′s national legacy and economic potential [13]. Herbal therapy plays a crucial role in healthcare in various fields, especially in regions where conventional medicine is inaccessible or costly, leading to an increasing demand for herbal treatments. Various bioactive plant compounds for medicinal properties have been thoroughly studied as a potential source of treatment options [14]. Historically, more than 60% of approved anticancer agents originate from plants or mimic bioactive compounds of plants [15]. For example, vincristine and vinblastine (vinca alkaloids), used in the treatment of leukemia, ovarian cancer, and breast cancer, among others, were derived from the periwinkle plant *Catharanthus roseus* [16]. In addition, paclitaxel, an anticancer drug used to treat breast cancer, was derived from the stem bark of the Pacific yew tree (*Taxus brevifolia*) [17]. Consequently, natural products, especially those obtained from plants, remain a valuable source for the development of new anticancer drugs. Phytochemicals integrate traditional medicine with modern medicinal development, establishing an extensive pool of innovative medicinal products. Medicinal plants discovered through traditional medicinal practices continue to direct the search for bioactive compounds, while advances in screening techniques and structural analysis have enhanced the isolation and characterization of these substances [18]. Ethnopharmacological records with in vitro pharmacological data have previously been used and partly validated in cancer models, which consistently demonstrated strong activity across diverse breast cancer cell lines [19]. The stem bark of *Tieghemella heckelii* (a tree species of the genus *Tieghemella*) found in Ghana has been exploited for its medicinal properties [20–22]. However, the chemical compounds found in distinct plant parts (roots, leaves, bark, flowers, fruits, and seeds) vary significantly among plant structures and consist of distinctive phytochemicals that have a variety of therapeutic properties, which suggests that some plant parts may be more useful than others in treating certain diseases [18]. Exploring diverse parts allows for a more comprehensive understanding of a plant′s medicinal potential, leading to more effective treatments.

Studies on the therapeutic properties of *T. heckelii* have mainly concentrated on the stem, bark, and seeds [20, 22]. However, it is important to investigate the anticancer potential of the leaves of *T. heckelii.* On a broader scale, research on the anticancer properties of *T. heckelii* has not been explored. Herbal anticancer therapies are a promising complement to traditional cancer treatments because of their therapeutic benefits. These natural products present an innovative approach to cancer treatment by enhancing treatment tolerance, improving quality of life, and potentially decreasing cancer progression. The goal of the study was to evaluate the in vitro anticancer activities of the leaves of *T. heckelii* on breast, prostate, and liver cancer cell lines. Anticancer medicinal properties of *T. heckelii* could be of global benefit by providing affordable and accessible treatment options, supporting traditional medical practices, promoting new drug development, and addressing the growing cancer burden. This is especially important considering the major issues faced globally, particularly in developing countries, in providing conventional cancer care, such as high costs, accessibility, and limited resources. This research provides some of the first available in vitro cytotoxicity data for *T. heckelii* leaves on these specific cell lines, as prior literature was largely nonexistent.

## 2. Materials and Methods

### 2.1. Sample Collection

The site of the location of *T. heckelii*, popularly named the “big tree,” has been developed into a tourist destination. It is in the Esen Epam Forest Reserve in Akim Oda (eastern region of Ghana) near the community called Asantemanso. *T. heckelii* species is considered rare and endangered due to overexploitation for timber. The tree is also found in the Bobiri Forest Reserve in the Ashanti Region as well. However, the tree located in Asantemanso is recognized as the only largest tree in West Africa. Standing at 66.5 m tall with a 12‐m circumference, this 400–500‐year‐old tree makes it a significant ecotourism site and historical landmark. It attracts visitors from various parts of the world, particularly from India and Asia, who come to perform rituals and seek spiritual assistance. The undamaged fresh leaves of *T. heckelii* were collected into a paper bag to prevent moisture buildup that could initiate microbial contamination.

### 2.2. Sample Transport and Preparation

The collected leaves were transported in a labeled (with sample name and type) paper bag to allow for drying and prevent condensation. The samples were transported the same day to the Clinical Pathology Department laboratory at Noguchi Memorial Institute for Medical Research, University of Ghana. Subsequently, the leaves of *T. heckelii* were presented to the herbarium of the Department of Plant and Environmental Biology, University of Ghana, for authentication.

### 2.3. Aqueous and Ethanolic Extraction of Plant Material

The leaves were thoroughly cleaned, air‐dried, and blended into a fine powder. This powder was subsequently divided into two batches, each weighing 50 g.

For the first batch, the powdered leaves were subjected to a cold maceration process in 500 mL of distilled water for 72 h. Following extraction, the resulting solution was filtered, frozen at −20°C, and then lyophilized (freeze‐dried) using a Labconco Freezone 6 Freeze Dryer (Missouri, United States) to produce the aqueous extract, coded AQTH.

The second batch of powdered leaves (50 g) was extracted with 500 mL of 70% ethanol for 72 h. After extraction, the solution was filtered, and the ethanol was evaporated from the filtered solution using a rotary evaporator (Ecodyst EcoChyll S, North Carolina, United States). The remaining aqueous portion was then frozen at −20°C and subsequently lyophilized (freeze‐dried) to yield the hydroethanolic extract, coded HETH. To test the solubility, 20 mg of the powdered extract was dissolved in 0.5 mL of 70% ethanol and distilled water for 30 min.

### 2.4. Reagents

Roswell Park Memorial Institute (RPMI)‐1640 medium, Dulbecco′s Modified Eagle Medium (DMEM), fetal bovine serum (FBS), penicillin–streptomycin (PS), trypsin, EDTA, and 3‐(4,5‐dimethylthiazol‐2‐yl)‐2,5‐diphenyltetrazolium bromide (MTT) were purchased from Sigma Chemical (Missouri, United States). All other reagents and chemicals used were of analytical grade and obtained from standard suppliers.

### 2.5. Cell Lines

#### 2.5.1. Breast Cancer (MDA‐MB‐468), Liver Cancer (HepG2), Prostate Cancer (PC3), and Vero Cell Lines

The cell lines were obtained from the RIKEN BioResource Center Cell Bank (Japan) in October 2024 and kept under cryopreservation, in liquid nitrogen (−196°C) for long‐term storage.

#### 2.5.2. Breast Cancer (MDA‐MB‐468) Cell Lines

The breast cancer cell line′s official name (MDA‐MB‐468) is an adenocarcinoma (cancerous epithelial tissue), specifically derived from a metastatic pleural effusion from a human (*Homo sapiens*) female patient, originating from the mammary gland (breast) tissue in a pleural effusion (a metastatic site). The Research Resource Identifier (RRID) for the human breast adenocarcinoma cell line MDA‐MB‐468 is RRID: CVCL_0419. The MDA‐MB‐468 (breast cancer) cell line is widely used as an in vitro model for biomedical research, primarily because it represents triple‐negative breast cancer (TNBC), an aggressive and hard‐to‐treat subtype. It provides a vital resource for evaluating novel prospective anticancer drugs.

#### 2.5.3. Human Hepatoblastoma (HepG2) Cell Lines

The official name HepG2 is the human hepatoblastoma cell line, derived from the liver tissue of a male with well‐differentiated epithelial carcinoma tissue of the liver (hepatoblastoma), initially described as hepatocellular carcinoma. The RRID for the HepG2 cell line is RRID: CVCL_0027. These cells are primarily utilized as an in vitro model for investigating human liver functions. It presents an important source of drug discovery on anticancer studies, as well as for toxicity studies, due to their vigorous proliferation in a laboratory environment.

#### 2.5.4. Human Prostate Carcinoma (PC3) Cell Lines

The official name for the PC3 cell line is human prostate carcinoma cell line derived from a bone metastasis of a Grade IV prostatic cancer in a male patient. It is a human prostate cancer (adenocarcinoma) cell line obtained from prostate tissue, specifically from bone metastases. The RRID for the PC3 cell line is RRID: CVCL_0035. Cell lines provide a significant in vitro model for investigating and assessing innovative therapeutic approaches, especially in anticancer research.

#### 2.5.5. Vero Cell Lines

The official name for Vero cell lines is Vero. Vero cell lines originated from the kidney epithelial tissue cells of a normal, adult female African green monkey (*Chlorocebus sabaeus*, formerly *Cercopithecus aethiops*). The general RRID for the Vero cell line is RRID: CVCL_0059. Vero cell lines mostly serve to evaluate a drug′s selectivity index (the ratio of a drug′s toxicity to its therapeutic effect on target cells) by using normal, noncancerous cells as an in vitro model to compare a compound′s toxic effects against cancerous cell lines.

#### 2.5.6. Cell Culture

Breast cancer (MDA‐MB‐468), liver cancer (HepG2), and normal kidney (Vero E6) cell lines were cultured in DMEM media, while prostate cancer (PC3) cell lines were cultured in RPMI medium. All media were supplemented with 10% FBS and 1% PS. Cells were kept in a humidified incubator at 37°C and 5% CO_2_.

#### 2.5.7. Cell Viability Assay

Cell viability was determined by the MTT method [23]. Briefly, cells (1 × 10^5^ cells/mL) were seeded in a 96‐well plate and incubated overnight to allow for cells to adhere. Cells were then treated with extracts at various concentrations (0–1000 *μ*g/mL) for 72 h. At the end of the incubation period, MTT solution (2.5 mg/mL) was added to each well, and the cells were incubated for another 4 h. The precipitated MTT‐formazan was dissolved in 0.04 N HCl–isopropanol, and the amount of formazan was measured at 595 nm using a microplate reader (Tecan M200 Pro, Japan). Cell viability was expressed as a percentage of the control culture.

#### 2.5.8. Statistical Analysis

All data were derived from at least three independent experiments. Data summarized using descriptive analysis, which looked at the mean and standard deviation (SD). Results were expressed as mean ± SD in all conditions. Cytotoxicity dose–response relationships were represented as a line graph and expressed as mean percent cell viability against different concentrations of the leaf extract of *T. heckelii*. Differences between groups were analyzed using Welch′s *t*‐tests (Bonferroni‐corrected), and a *p* < 0.05 was considered statistically significant.

## 3. Results (MDA, HepG2, and PC3)

The anticancer activity of the extracts was evaluated in vitro against human liver cancer (HepG2), breast cancer (MDA‐MB‐468), and prostate cancer (PC3), as well as Vero E6 (African green monkey kidney) as a normal cell line to evaluate the safety of the plant extracts.

The present study showed that both aqueous and ethanolic extracts had promising cytotoxic activities on all cell lines tested. All the extracts exhibited a dose‐dependent decrease in cell viability (Figure 1), with the PC3 cell line showing the most pronounced response.

Figure 1Cytotoxic activity of the extracts and curcumin standard on (a) HepG2 human liver cancer cell line, (b) MDA‐MB‐468 human breast cancer cell line, (c) PC3 human prostate cancer cell line, and (d) Vero African green monkey kidney normal cell line. The graph shows the cell viability (%) of cells treated with a range of concentrations (10–200 *μ*g/mL) of extracts and curcumin (standard) for 72 h, as determined by the MTT assay. Values are represented as means ± SD of three replicates.

**Figure:**
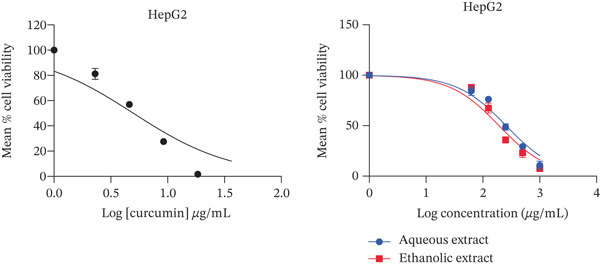
(a)

**Figure:**
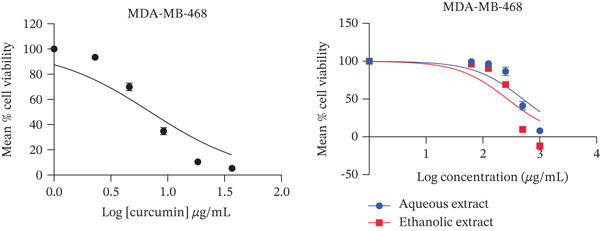
(b)

**Figure:**
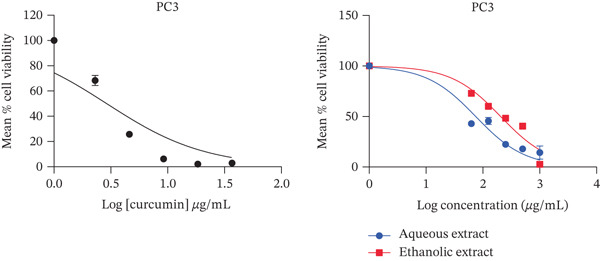
(c)

**Figure:**
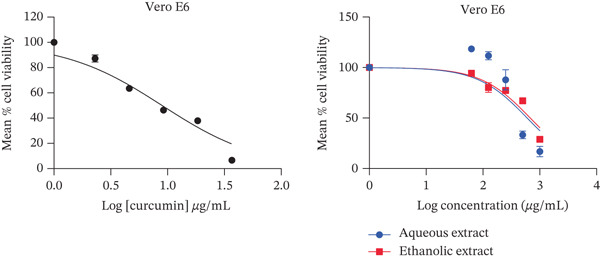
(d)

Both ethanolic and aqueous extracts demonstrated similar cytotoxicity for the HepG2 cell line, with IC_50_ values of about 200 *μ*g/mL. Selectivity index of > 2 was recorded by all cell lines with IC_50_ values of 74–506 *μ*g/mL. The selectivity index of the ethanolic extract was higher than 2 (Table 1). When compared to other cell lines, the aqueous extract for prostate cancer showed the lowest IC_50_ value with a selectivity index of 8 (Table 1). In addition, the extracts showed less cytotoxic activity against the normal (Vero) cell line.

**Table 1:** IC_50_ of aqueous and ethanolic extracts on human cancer cell lines and a normal (Vero E6) cell line.

	HepG2	MDA‐MB‐468	PC3	Vero E6
Curcumin	4.991 ± 0.19	7.044 ± 0.19	2.909 ± 0.47	8.95 ± 0.26
Aqueous extract	255.5 ± 16.71	506.9 ± 30.94	74.64 ± 2.01	599.6 ± 20.56
Ethanolic extract	192.5 ± 10.38	271.1 ± 6.53	204.9 ± 16.33	679.9 ± 16.18

*Note:* IC_50_ results are expressed in micrograms per milliliter as mean ± SD (*n* = 3). The 95% CIs were within ±15% of the mean. Welch′s *t*‐tests (Bonferroni‐corrected) showed the following: PC3, aqueous more potent than ethanolic (*p* < 0.01); HepG2: ethanolic < aqueous (*p* < 0.05); MDA‐MB‐468: ethanolic < aqueous (*p* < 0.01). Both extracts were significantly less potent than curcumin (*p* < 0.001).

The selectivity index is the ratio of the IC_50_ values of the treatments on normal cells to the IC_50_ for the respective cancer cell lines. The selectivity of compounds against cancer cell lines was confirmed by testing their cytotoxicity on Vero (African green monkey kidney) normal cells. Overall, the extracts showed good selectivity of > 2 on the cell lines except for MDA‐MB‐468, where the aqueous extract showed a selectivity of < 2 (Table 2). The aqueous extract exhibited good activity against the PC3 cell line with an IC_50_ of < 100 *μ*g/mL (Table 1) and was highly selective (SI = 8.03) for the PC3 cells. Both extracts showed good anticancer activity against the HCT‐15 cell line with good selectivity.

**Table 2:** Selectivity index (SI) of extracts.

	HepG2	MDA‐MB‐468	PC3
Curcumin	1.79	1.27	3.08
Aqueous extract	2.35	1.18	8.03
Ethanolic extract	3.53	2.51	3.32

*Note:* The selectivity index is the ratio of the IC_50_ values of the treatments on normal cells to the IC_50_ for the respective cancer cell lines. SI values are mean ± 95*%*CI. Aqueous extract showed the highest selectivity for PC3 (SI = 8.0), while ethanolic extract was most selective for HepG2 (SI = 3.5).

## 4. Discussion

The present study provides the first evidence, to our knowledge, of the cytotoxic potential of *T. heckelii* leaf extracts against breast (MDA‐MB‐468), liver (HepG2), and prostate (PC3) cancer cell lines, with promising selectivity over normal (Vero E6) cells. Both aqueous and ethanolic extracts demonstrated dose‐dependent inhibition of cancer cell proliferation, although their potency and selectivity varied across cell types. These findings suggest that *T. heckelii* leaves harbor bioactive constituents with anticancer potential that merit further investigation.

Our results highlight the remarkable activity of the aqueous extract against PC3 (prostate cancer) cells (IC_50_ = 74.64 * μ*g/mL; SI = 8.03), indicating both potency and strong selectivity. According to the US National Cancer Institute (NCI) guidelines, an IC_50_ ≤ 30 * μ*g/mL is considered highly active, whereas values below 100 *μ*g/mL are still regarded as biologically meaningful for crude extracts [24, 25] Thus, the activity observed against PC3 cells is notable, especially given the extract′s low toxicity toward normal kidney cells. This aligns with growing evidence that plant‐derived compounds can offer safer, targeted cytotoxicity against prostate cancer compared to conventional chemotherapeutics, which are often limited by systemic toxicity [26, 27].

The ethanolic extract also demonstrated appreciable activity, with IC_50_ values of 192.5 *μ*g/mL (HepG2), 271.1 *μ*g/mL (MDA‐MB‐468), and 204.9 *μ*g/mL (PC3). Interestingly, its selectivity index was highest for HepG2 cells (SI = 3.53), suggesting that the ethanolic preparation may contain compounds preferentially toxic to liver cancer cells. Several studies have shown that solvent polarity influences phytochemical extraction profiles, with ethanol often enriching for flavonoids, alkaloids, and terpenoids known for anticancer activities [28–32]. The differential cytotoxicity observed between aqueous and ethanolic extracts may therefore reflect variation in the solubility and concentration of active metabolites.

When compared to the positive control curcumin (IC_50_ values ranging 2.9–7.0 *μ*g/mL), both extracts were less potent but still showed meaningful selectivity indices (> 2 in most cases). This is important, as crude extracts are not expected to match the potency of purified compounds; rather, they serve as a source of leads for subsequent bioassay‐guided fractionation and compound isolation [33]. The selectivity indices observed (> 3 in HepG2 and PC3 for ethanolic extract and > 8 for PC3 with aqueous extract) are within or above the thresholds considered favorable for drug discovery [34].

From a pharmacological perspective, the pronounced selectivity against PC3 cells is particularly intriguing. Prostate cancer remains a major global health burden, and resistance to conventional therapies necessitates new agents with novel mechanisms of action [35]. The phytoconstituents of *T. heckelii* may target unique molecular pathways relevant to prostate cancer survival, possibly involving apoptosis induction, cell cycle arrest, or oxidative stress pathways, as has been documented for other African medicinal plants [36, 37].

Anticancer properties in plants are often distributed throughout the entire parts of the same plants, including their leaves and stems [38]. The present study reported the anticancer potential of the leaves of *T. heckelii* and concurs with the study [39], which reported the potential anticancer activity of the stembark of *T. heckelii*.

The anticancer potential of *T. heckelii* is likely driven by the synergy of triterpenoid saponins and phenolic compounds. Saponins from *T. heckelii*, such as *tieghemelin A*, have been shown to induce apoptosis (IC_50_ 13.5 *μ*M) (apoptosis induction via the upregulation of Bax and downregulation of Bcl‐2, triggering the mitochondrial pathway) [39]. Polyphenols such as quercetin and luteolin (found in *T. heckelii*) are known to cause cell cycle arrest in the G1 phase by inhibiting cyclin‐dependent kinases (CDK1) [39]. The extracts have been reported to have significant antioxidant capacity, which could potentially help in the disruption of the pro‐oxidant conditions required for tumor proliferation by targeting SOD and GPx levels [39]. Leaf extracts from various medicinal plants have been shown to have significant cytotoxic effects on PC3 (prostate cancer) cells when compared to other plant parts [40]. Primarily, this occurs by inducing apoptosis, causing significant apoptotic nuclear variations, such as condensation, fragmentation, and modulating the cell cycle.

Although this study provides promising in vitro data, several limitations must be acknowledged. First, crude extracts contain a complex mixture of compounds, and the precise bioactive molecules remain unidentified. Second, in vitro cytotoxicity does not always translate directly to in vivo efficacy due to factors such as metabolism, bioavailability, and tumor microenvironment. Third, the use of a single normal (Vero E6) cell line provides a limited assessment of safety. Also, mechanistic studies employing apoptosis markers, caspase activity, and transcriptomic profiling would be valuable in elucidating this pathway, which warrants further studies.

## 5. Conclusion

In conclusion, our findings demonstrate that leaves of *T. heckelii* possess cytotoxic properties with notable selectivity against prostate (PC3) and liver (HepG2) cancer cell lines. These results provide a scientific basis for the traditional use of *T. heckelii* and position it as a potential source of novel anticancer agents. By advancing from crude extracts to purified bioactive compounds, *T. heckelii* could contribute to the discovery of safe and effective plant‐derived anticancer therapeutics. Therefore, further investigation is recommended to confirm the bioactive compounds responsible for the anticancer activity, which will help determine the potential active components and contribute to understanding their mechanisms of action. This is an in vitro study and therefore has the inability to properly mimic the complex environment of a living organism, difficulty in simulating long‐term impacts and full‐body responses, and the inability to accurately predict in vivo activity.

Future studies should therefore focus on bioassay‐guided fractionation to isolate and characterize the active properties and assess their molecular mechanisms, and evaluate their efficacy and toxicity in relevant in vivo cancer models.

## Author Contributions

Justice Kumi conceptualized the study and was involved in methodology, sample collection, data analysis, supervision, writing the original draft, and reviewing and editing of the final draft. Eric N. Y. Nyarko and Abigail Aning were involved in laboratory analysis, methodology, data curation, and writing, review, and editing of the final draft. Janet Ampofo and Sherif Hamidu contributed to the writing, reviewing, and editing of the final draft.

## Funding

No funding was received for this manuscript.

## Disclosure

All the authors have read and approved the final manuscript for publication.

## Conflicts of Interest

The authors declare no conflicts of interest.

## Data Availability

The data that support the findings of this study are available from the corresponding authors upon reasonable request.
